# Paired box 8 suppresses tumor angiogenesis and metastasis in gastric cancer through repression of FOXM1 via induction of microRNA-612

**DOI:** 10.1186/s13046-018-0830-3

**Published:** 2018-07-18

**Authors:** Liyan Wang, Xiaotong Bo, Qinghua Zheng, Wenhong Ge, Yanhua Liu, Bin Li

**Affiliations:** Department of Digestive Diseases, Affiliated Hospital of Guilin Medical University, No. 15, Lequn Road, Guilin, 541001 China

**Keywords:** Angiogenesis, EMT, Gastric cancer, Invasion, PAX8

## Abstract

**Background:**

Paired box 8 (PAX8) has been documented to be downregulated in gastric cancer. However, its biological function in this malignancy is poorly understood.

**Methods:**

In the present work, we investigated the effects of PAX8 overexpression and knockdown on the aggressive phenotype of gastric cancer cells. We further checked the involvement of forkhead box M1 (FOXM1), a ubiquitously expressed oncogene that can facilitate gastric cancer progression, in the action of PAX8.

**Results:**

Ectopic expression of PAX8 blocked the migration and invasion of both AGS and SGC-7901 cells, but had no effect on cell proliferation. Conversely, knockdown of PAX8 enhanced gastric cancer cell migration and invasion. PAX8 overexpression inhibited epithelial-mesenchymal transition (EMT) and pro-angiogenic activity of gastric cancer cells. Mechanistically, PAX8 overexpression downregulated FOXM1 by stimulating microRNA (miR)-612 expression. Ectopic expression of miR-612 recapitulated the effect of PAX8 overexpression on gastric cancer cells, causing an inhibition of migration, invasion, EMT, and angiogenesis. Knockdown of miR-612 or overexpression of FOXM1 significantly reversed the tumor-suppressive activity of PAX8. In vivo studies further demonstrated that PAX8 overexpression restrained tumor angiogenesis and metastasis in nude mice, which was accompanied by increased expression of miR-612 and decreased expression of FOXM1.

**Conclusions:**

PAX8 exerts a tumor-suppressive effect against gastric cancer cells, largely through induction of miR-612 and repression of FOXM1. Therefore, restoration of PAX8 expression may offer therapeutic benefits in the treatment of gastric cancer.

## Background

Paired box (PAX) transcription factors encoded by developmental control genes share a highly conserved paired-box DNA-binding domain (PD). At present, nine Pax genes (Pax1 to Pax9) have been identified in humans [1]. PAX proteins have the ability to mediate DNA binding and transcriptional activation through the PD and homeodomain (HD) [2]. Compelling evidence indicates the link between Pax dysregulation and cancer progression [3, 4]. Overexpression of Pax3 leads to enhanced osteosarcoma metastasis [3]. Pax4 can promote the invasion and metastasis of epithelial cancers by inhibiting the expression of microRNA (miR)-144 and miR-451 [5]. miRs are thought to act as a negative regulator of a large number of genes through interaction with the 3′-untranslated regions (3’-UTR) of target mRNAs [6]. Pax8 is aberrantly expressed in several cancer types including papillary thyroid carcinoma [7], cervical tumors [8], and glioblastoma [9]. Overexpression of PAX8 has a poor prognostic impact on patients with endometrial cancer [10]. PAX8 is required for the aggressive phenotype of ovarian cancer cells, as evidenced by the finding that silencing of Pax8 significantly decreased cell proliferation, migration, and invasion [11]. In contrast to ovarian cancer, gastric cancer shows little or no expression of PAX8 [12], suggesting conflicting roles of PAX8 in cancer progression.

Forkhead box M1 (FOXM1), a member of the Fox family, functions as an oncogene in multiple cancers such as glioma and lung cancer [13, 14]. FOXM1 overexpression has been documented to facilitate the migration and invasion of gastric cancer cells via induction of Cathepsin D [15]. miR-630-mediated downregulation of FOXM1 can inhibit the epithelial-to-mesenchymal transition (EMT) of gastric cancer cells [16]. Knockdown of FOXM1 increases the sensitivity of gastric cancer cells to cisplatin [17]. These studies support that FOXM1 is an important therapeutic target for gastric cancer.

In the present study, we explored the biological role of PAX8 in the growth and metastasis of gastric cancer. In addition, the underlying molecular mechanism was investigated.

## Methods

### Tissue specimens

Nineteen paired gastric cancer and adjacent noncancerous gastric tissue samples were collected from gastric cancer patients who received surgical resection at our hospital between April 2015 and November 2016. Freshly resected tissue samples were snap-frozen and stored in liquid nitrogen until gene expression analysis. All cases were diagnosed histologically, and none of them underwent preoperative chemotherapy or radiotherapy. Written informed consent for research purpose was obtained from each patient. This study was approved by the Ethics Committee of Guilin Medical University (Guilin, China).

### Cell culture and treatment

Human gastric cancer cell lines (AGS, SGC-7901, MKN-28, and MKN-45) and immortalized human gastric epithelial GES-1 cells were obtained from the Institute of Biochemistry and Cell Biology, Chinese Academy of Sciences (Shanghai, China). Cells were maintained in RPMI 1640 medium (Invitrogen, Carlsbad, CA, USA) supplemented with 10% fetal bovine serum (FBS; Sigma-Aldrich, St. Louis, MO, USA). Human umbilical vein endothelial cells (HUVECs) were purchased from ScienCell Research Laboratories (Carlsbad, CA, USA) and maintained in Endothelial Cell Medium (ScienCell Research Laboratories) containing 10% FBS.

### Western blot analysis

Cells and tissue samples were lysed in ice-cold radioimmunoprecipitation assay buffer supplemented with the Protease Inhibitor Cocktail (Sigma-Aldrich). The lysates were resolved in sodium dodecyl sulfate polyacrylamide gels and transferred onto polyvinylidene fluoride membranes. The membranes were blocked with 5% fat-free milk and incubated with anti-PAX8, anti-E-cadherin, anti-vimentin, anti-vascular endothelial growth factor (VEGF), anti-FOXM1, anti-FOXC2, anti-FOXF1, anti-FOXL1, and anti-β-actin (Abcam, Cambridge, MA, USA) at 4 °C overnight. The membranes were then incubated with horseradish peroxidase-conjugated secondary antibody for 1 h. Signals were developed by enhanced chemiluminescence (Merck Millipore, Darmstadt, Germany).

### Real-time PCR analysis

For quantification of miR expression, total RNA was extracted from tissues and cells using Trizol reagent (Invitrogen) and reversely transcribed using the TaqMan miRNA Reverse Transcription Kit (Applied Biosystems, Foster City, CA, USA). The expression of miR-612, miR-877-5p, miR-920, and miR-423-5p was examined by real-time PCR analysis using the TaqMan MicroRNA Assay Kit (Applied Biosystems). RUN48 was used as an endogenous control. For measurement of PAX8 mRNA abundance, total RNA was subjected to reverse transcription using SuperScript III First-Strand Synthesis System and random primers (Invitrogen). Real-time PCR analysis of PAX8 mRNA was performed using the SYBR Green dye-based detection system (Applied Biosystems) with the following primers: forward, 5′-TTTGCTTGGCTCTTTCTACACCTC-3′; reverse, 5′-GAATGTCTGTTTTAAGCTCCCTGG-3′ [18]. GAPDH was used as a normalization control.

### Plasmid construction and transfection

Human PAX8 and FOXM1 cDNA (lacking the 3’-UTR) was purchased from OriGene Technologies (Rockville, MD, USA) and cloned into pcDNA3.1(+). The constructs were verified by sequencing. miR-612 mimic, anti-miR-612 inhibitor, and their negative controls were purchased from Thermo Scientific, Lafayette, CO, USA). PAX8-targeting small interfering RNA (siRNA) and negative control siRNA were purchased from Sigma-Aldrich. Cell transfections were performed using Lipofectamine 3000 reagent (Invitrogen) as per the manufacturer’s instructions. miR-612 mimic, anti-miR-612 inhibitor, and siRNAs were transfected at a final concentration of 40 nM.

### Cell proliferation assay

Cells were seeded onto 96-well plates (3000 cells/well) and tested for viability every 12 h until 72 h. The 3-(4,5-dimethylthiazol-2-yl)-2,5-diphenyltetrazolium bromide (MTT) solution (0.5 mg/mL; Sigma-Aldrich) was added to each well and incubated for 4 h at 37 °C. The spectrophotometric absorbance of each well was measured at 570 nm.

### In vitro wound-healing assay

Cells were seeded onto 6-well plates and allowed to grow to confluence. After serum starvation for 24 h, a sterile 200-μL pipette tip was used to generate an artificial wound in the cell monolayer. Cells were cultured for 24 h in the presence of mitomycin C (10 μg/mL; Sigma-Aldrich), which was used to inhibit cell proliferation. The percentage of wound closure was determined from three independent experiments.

### Transwell invasion assay

Transwell chambers (8 μm in pore size) were used to assess cell invasion. Briefly, 3 × 10^4^ cells suspended in serum-free media were seeded onto the upper chamber of 24-well plates, which was precoated with Matrigel (BD Pharmingen, San Jose, CA, USA) overnight. The lower chamber was filled with cell culture media containing 10% FBS. After incubation for 24 h, the cells that invaded through the Matrigel membrane were fixed, stained with 0.1% crystal violet (Sigma-Aldrich), and counted under a phase-contrast microscope.

### Conditioned medium from tumor cells

Gastric cancer cells transfected with indicated constructs were seeded onto 6-well plates (1 × 10^6^ cells/well) and cultured in complete medium to reach confluence. The medium was replaced by serum-free medium. Following incubation for another 24 h, the conditioned medium was harvested and filtered through a 0.45-μm membrane.

### In vitro endothelial cell tube formation assay

In vitro endothelial cell tube formation assay was carried out as described previously [19]. Briefly, HUVECs (4 × 10^4^ cells/well) were plated onto 24-well plates precoated with growth factor-reduced Matrigel (BD Pharmingen) and cultured in conditioned media for 16 h. Capillary-like structures were photographed, and cumulative tube length was calculated.

### Enzyme-linked immunosorbent assay (ELISA)

Human VEGF ELISA Kit (R&D Systems, Minneapolis, MN, USA) was used to determine the concentration of VEGF in conditioned media from gastric cancer cells transfected with indicated constructs.

### Animal studies

The experiments involving animals were approved by the Ethics Committee for the Use and Care of Animals of Guilin Medical University (Guilin, China). SGC-7901 cells stably expressing PAX8 or empty vector were injected through the tail vein of male BALB/C nude mice (5 week old; 4 × 10^6^ cells/mouse). Seven weeks later, the mice were sacrificed. The lung tissues were harvested and photographed. Some of lung tissue samples were fixed and processed for immunostaining for CD31 using a polyclonal anti-CD31 antibody (Abcam). The others were subjected to Western blot analysis. For assessment of tumor angiogenesis [20], most vascular areas (so called hot-spots) were located at low magnification and CD31-positive microvessels were counted on a 200× magnification field. Mean vessel density (MVD) was determined based on 4 microscopic fields.

### Statistical analysis

Quantitative data are expressed as means ± standard deviation. The statistical significance between groups was evaluated using the Student’s *t* test or one-way analysis of variance (ANOVA) followed by the Tukey test. A *P* < 0.05 was considered statistically significant.

## Results

### PAX8 inhibits the migration and invasion of gastric cancer cells in vitro

**Fig. 1:**
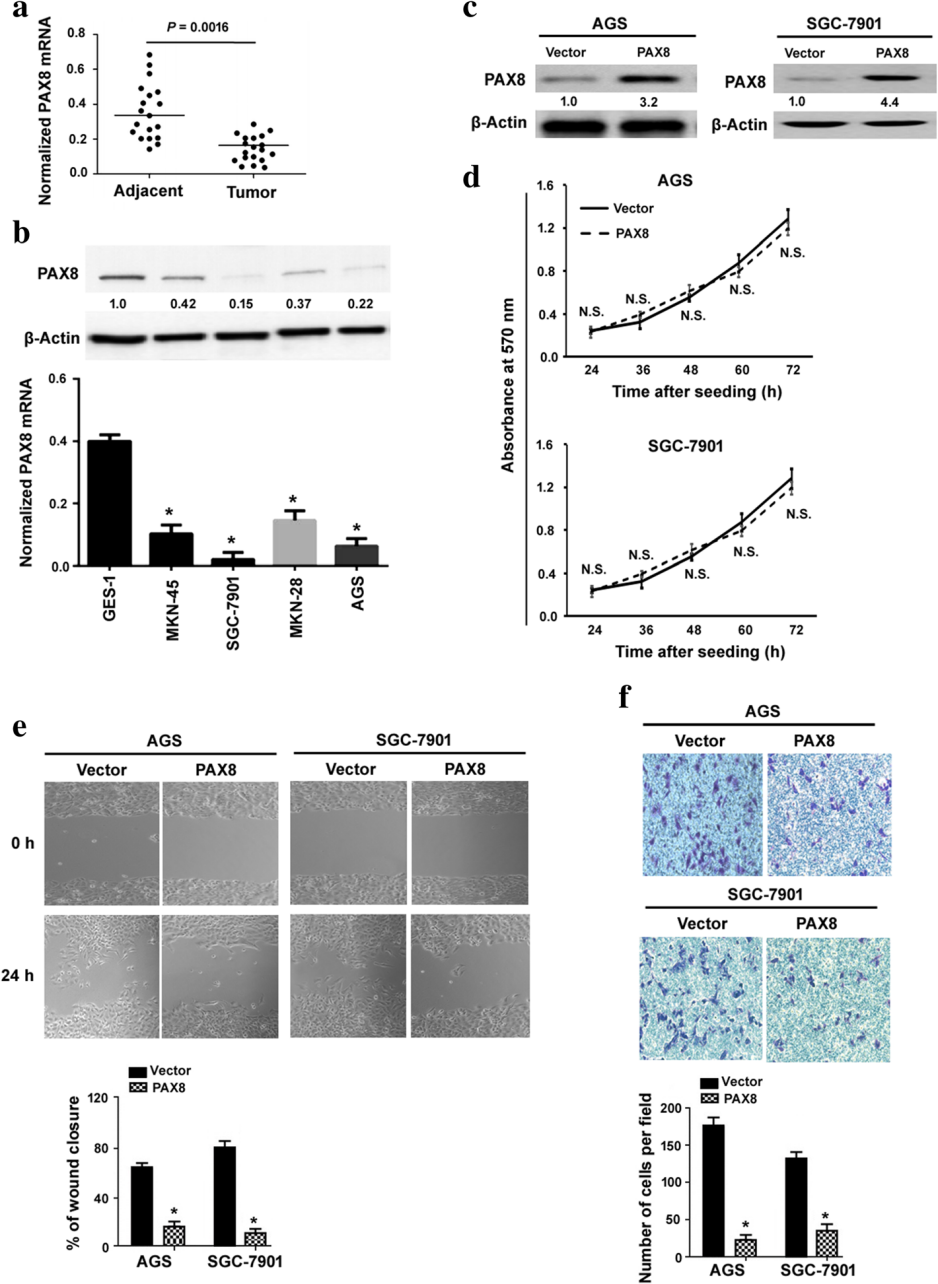
PAX8 inhibits the migration and invasion of gastric cancer cells in vitro. **a** Real-time PCR analysis of PAX8 mRNA levels in 19 pairs of gastric cancer and adjacent noncancerous tissues. **b** Analysis of PAX8 protein (upper) and mRNA (lower) expression in indicated cell lines by real-time PCR and Western blotting, respectively. Numbers below Western blots indicate fold change relative to the value in GES-1 cells. ^*^P < 0.05 vs. GES-1 cells. **c** Western blot analysis of PAX8 protein levels in AGS and SGC-7901 cells transfected with PAX8-expressing plasmid or empty vector. **d** Measurement of the proliferation of AGS and SGC-7901 cells transfected with PAX8-expressing plasmid or empty vector after culturing for 48 and 72 h. N.S. indicates no significance. **e** In vitro wound-healing assay was performed to assess the migrative capacity of AGS and SGC-7901 cells transfected with PAX8-expressing plasmid or empty vector. ^*^P < 0.05 vs. vector-transfected cells. **f** Transwell invasion assay was used to determine the invasive ability of AGS and SGC-7901 cells transfected with PAX8-expressing plasmid or empty vector. ^*^P < 0.05 vs. vector-transfected cells

It has been reported that PAX8 expression is weak or absent in gastric cancer [12]. To confirm the expression of PAX8 in gastric cancer, we examined the mRNA and protein expression of PAX8 in 19 pairs of gastric cancer and adjacent noncancerous gastric tissues. Quantitative real-time PCR assay revealed that PAX8 mRNA levels were significantly lower in gastric cancer than those in adjacent noncancerous tissues (*P* = 0.0016; Fig. 1a). Compared to GES-1 gastric epithelial cells, the expression level of PAX8 was significantly reduced in multiple gastric cancer cell lines including AGS, SGC-7901, MKN-28, and MKN-45 (Fig. 1b). These results indicate that PAX8 is downregulated in gastric cancer.

**Fig. 2:**
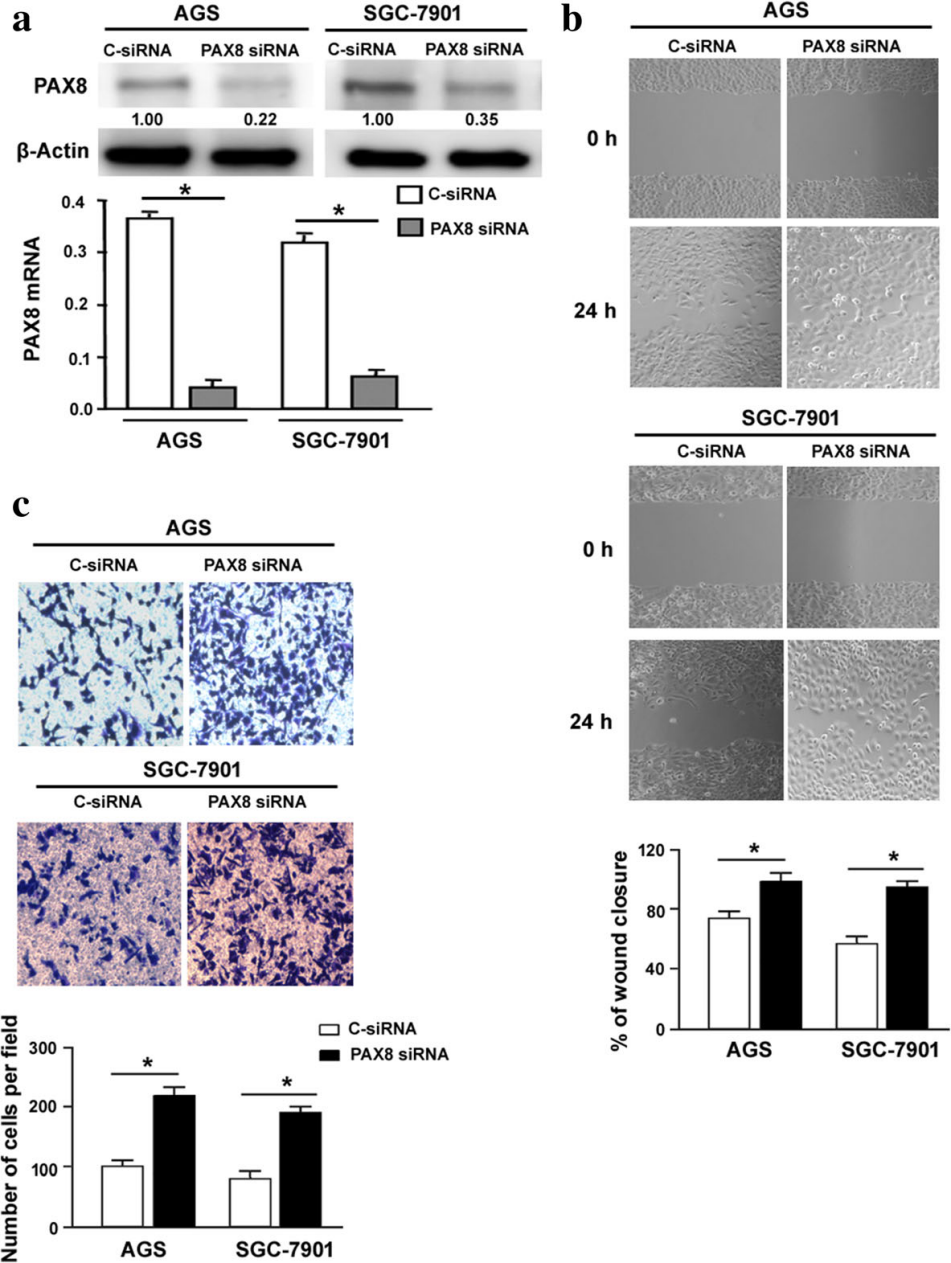
Knockdown of PAX8 promotes gastric cancer cell migration and invasion. **a** The levels of PAX8 transcripts were decreased in cells transfected with PAX8-targeting siRNA. **b** In vitro wound-healing assay was performed to assess the migrative capacity of AGS and SGC-7901 cells transfected with PAX8-targeting or control siRNA. **c** Transwell invasion assay was used to determine the invasive ability of AGS and SGC-7901 cells transfected with PAX8-targeting or control siRNA. ^*^P < 0.05

To explore the biological function of PAX8, we overexpressed PAX8 in both AGS and SGC-7901 cells. As determined by Western blot analysis, the protein levels of PAX8 were markedly increased in AGS and SGC-7901 cells transfected with PAX8 (Fig. 1c). MTT assay revealed that ectopic expression of PAX8 did not affect the number of viable cells at each time point tested (Fig. 1d). Of note, overexpression of PAX8 significantly reduced cell migration (Fig. 1e) and invasion (Fig. 1f) in AGS and SGC-7901 cells. Conversely, knockdown of PAX8 (Fig. 2a) led to a significant enhancement of cell migration (Fig. 2b) and invasion (Fig. 2c).

### PAX8 suppresses EMT and angiogenic activity in gastric cancer cells

**Fig. 3:**
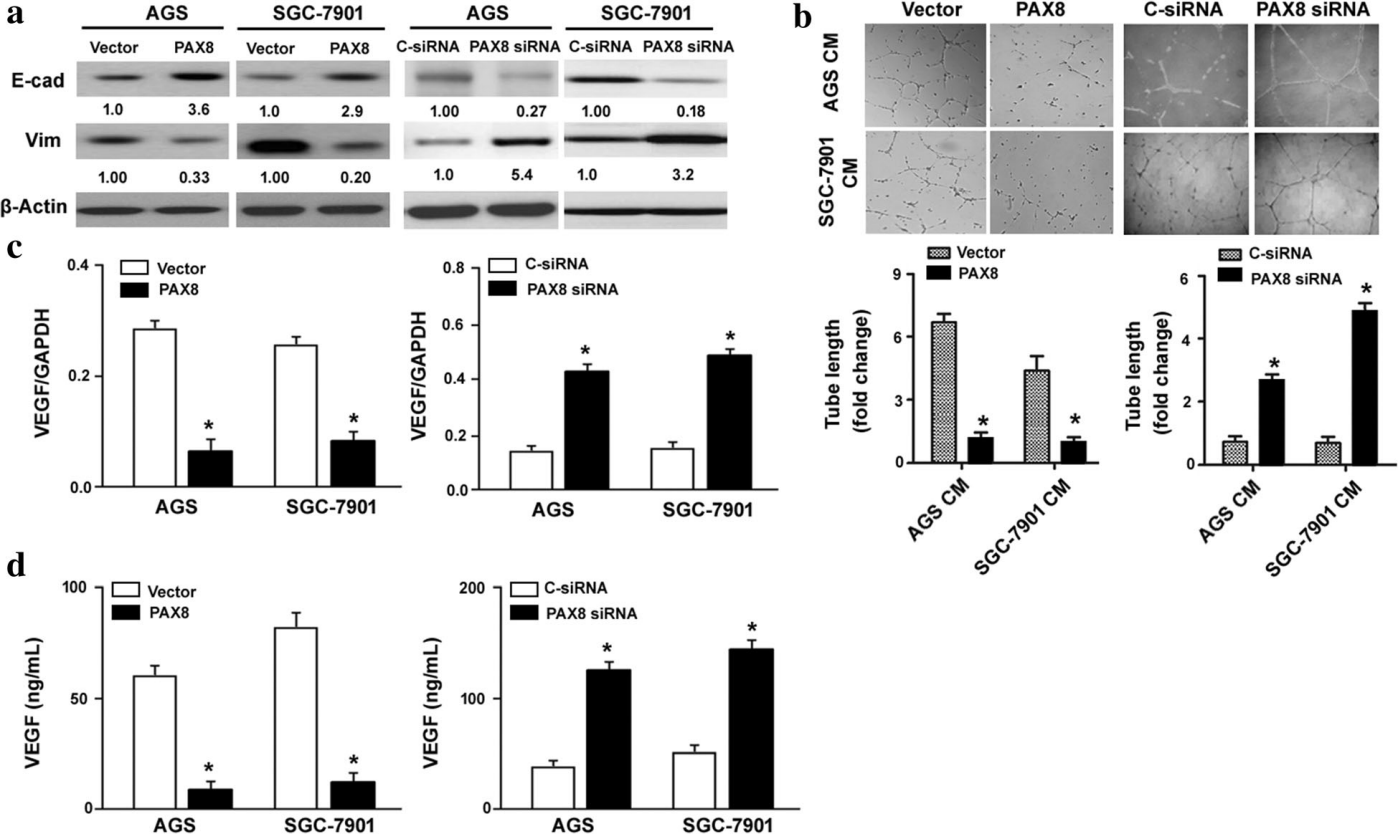
PAX8 suppresses EMT and angiogenic activity in gastric cancer cells. **a** Western blot analysis of E-cadherin (E-cad) and vimentin (Vim) protein levels. **b** In vitro endothelial cell tube formation assay revealed that conditioned media from PAX8-overexpressing AGS and SGC-7901 cells significantly suppressed formation of tube-like structure by HUVECs. **c** Real-time PCR analysis of VEGF mRNA levels. **d** Measurement of VEGF concentrations in conditioned media from PAX8-overexpressing and control AGS and SGC-7901 cells by ELISA. ^*^P < 0.05 vs. vector-transfected cells

Next, we examined the effect of PAX8 overexpression on EMT and angiogenic of gastric cancer cells. EMT has been linked to enhanced invasive ability in many cancer cell types [21, 22]. Western blot analysis showed that AGS and SGC-7901 cells stably expressing PAX8 had a marked increase in the expression of E-cadherin (an epithelial marker) and decrease in the expression of vimentin (a mesenchymal marker) (Fig. 3a), suggesting an inhibition of EMT. In vitro endothelial cell tube formation assay revealed that conditioned media from PAX8-overexpressing AGS and SGC-7901 cells significantly suppressed tube-like structure formation (*P* < 0.05; Fig. 3b). Consistent with this, VEGF expression and secretion was significantly reduced in PAX8-overexpressing AGS and SGC-7901 cells, compared to empty vector-transfected counterparts (*P* < 0.05; Fig. 3c and d). In contrast, PAX8 downregulation reduced the expression of E-cadherin and increased the expression of vimentin (Fig. 3a). PAX8 silencing led to an increase in VEGF production (Fig. 3c and d) and enhanced the angiogenic activity (Fig. 3b) of gastric cancer cells.

### PAX8 downregulates FOXM1 in gastric cancer cells by inducing miR-612

**Fig. 4:**
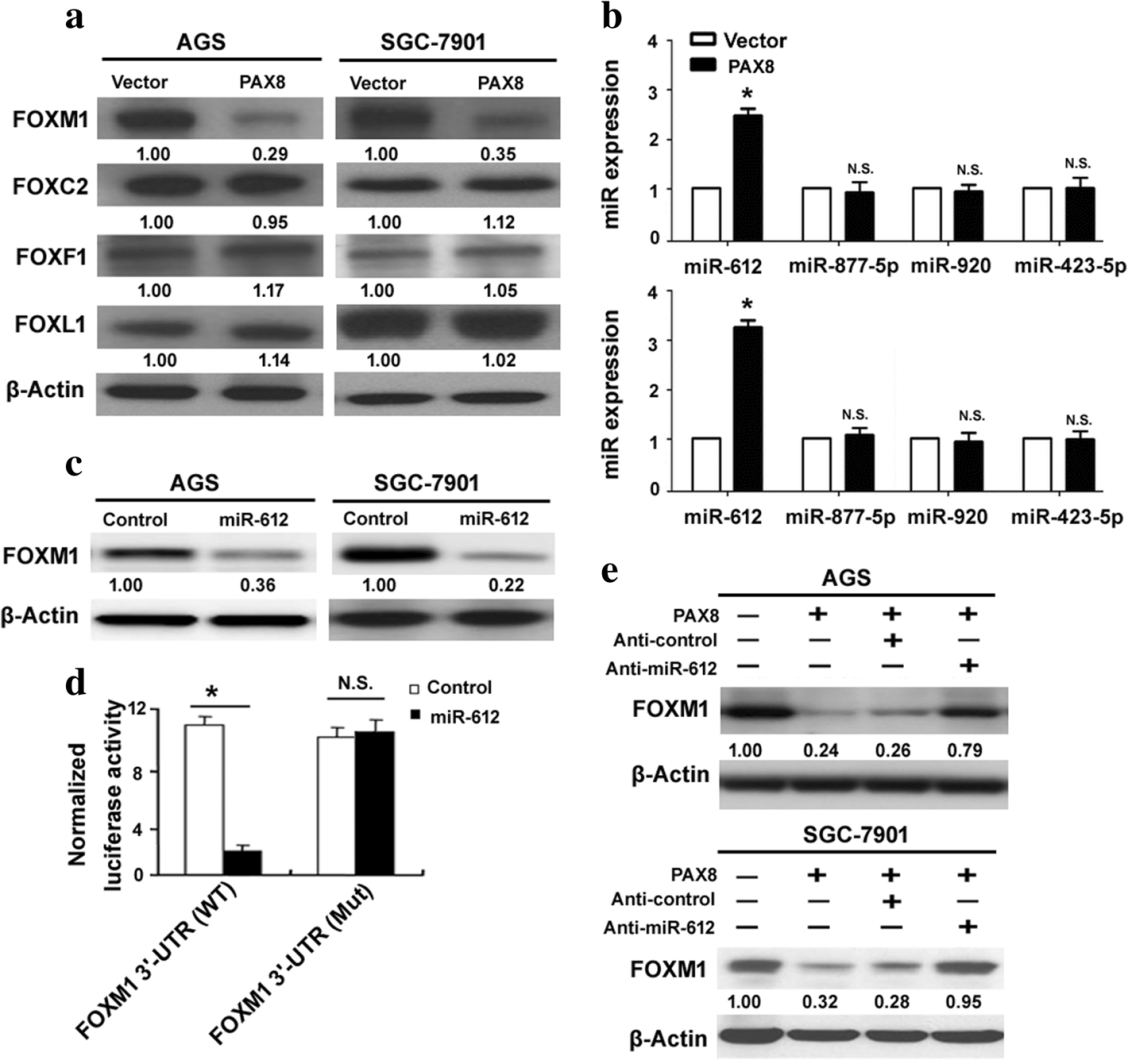
PAX8 stimulates miR-612 to downregulate FOXM1 in gastric cancer cells. **a** Western blot analysis of indicated proteins in AGS and SGC-7901 cells transfected with PAX8-expressing plasmid or empty vector. **b** Real-time PCR analysis of indicated miRs. ^*^P < 0.05 vs. vector-transfected cells. N.S. indicates no significance. **c** Analysis of FOXM1 protein levels in AGS and SGC-7901 cells transfected with control miR or miR-612. **d** Knockdown of miR-612 prevented the inhibition of FOXM1 by PAX8, as determined by Western blot analysis

Several lines of evidence have supported that FOXM1 acts as a promoter of angiogenesis and metastasis in gastric cancer [15, 16]. Of note, ectopic expression of PAX8 led to 3–6-fold reduction in the FOXM1 protein level in both AGS and SGC-7901 cells (Fig. 4a). In contrast, multiple other FOX proteins including FOXC2, FOXF1, and FOXL1 were not altered by PAX8 overexpression (Fig. 4a). These results indicate the selective inhibition of FOXM1 by PAX8 in gastric cancer cells.

Next, we searched for potential miR mediator(s) in the negative regulation of FOXM1 by PAX8. We predicted many miR regulators of FOXM1 based on the miRDB algorithm (data not shown). As determined by real-time PCR analysis, miR-612 but not miR-877-5p, miR-920, or miR-423-5p was upregulated in PAX8-overexpressing AGS and SGC-7901 cells (Fig. 4b). Notably, transfection with miR-612 mimic led to a significant decline in the FOXM1 protein levels in both AGS and SGC-7901 cells (Fig. 4c). Luciferase reporter assay further confirmed that miR-612 directly bound to the 3’-UTR of FOXM1 mRNA, causing the repression of the luciferase reporter construct (Fig. 4d). In addition, knockdown of miR-612 markedly prevented the inhibition of FOXM1 by PAX8 (Fig. 4e). Collectively, induction of miR-612 accounts for PAX8-mediated downregulation of FOXM1.

### miR-612/FOXM1 axis is involved in the tumor-suppressive activity of PAX8 in gastric cancer cells

**Fig. 5:**
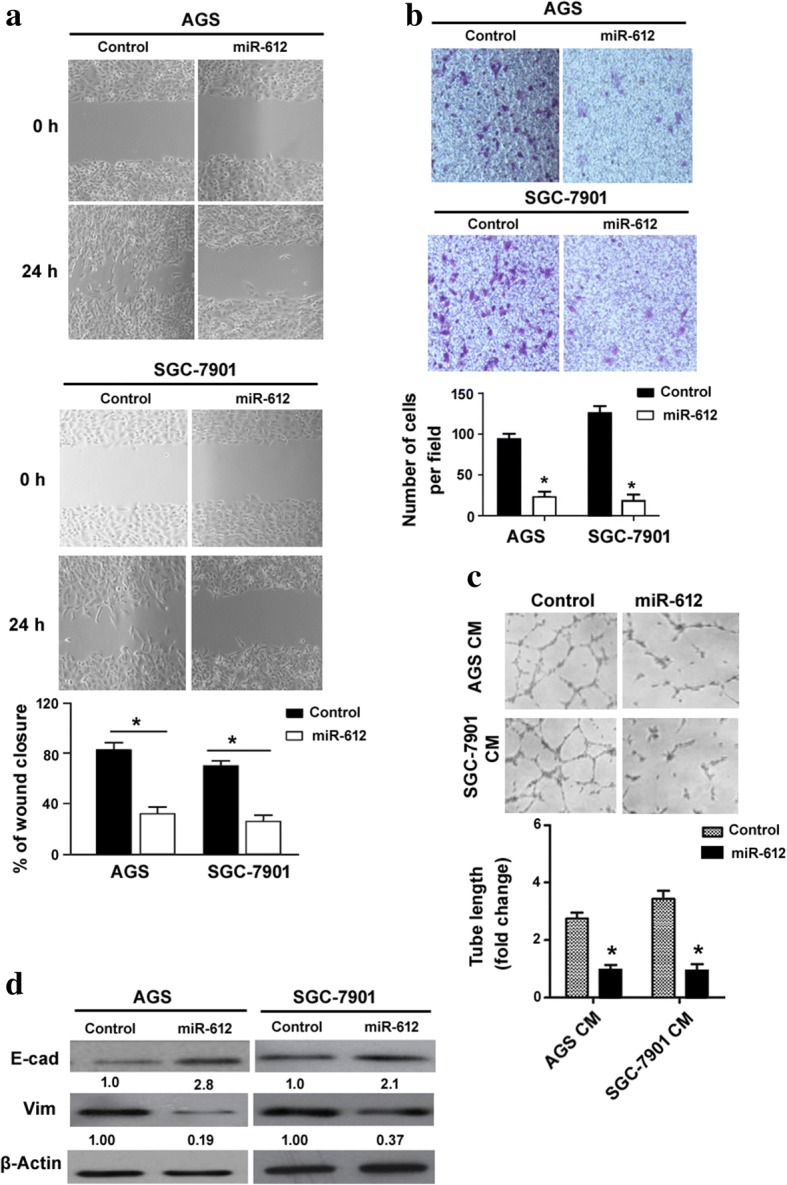
miR-612 exerts suppressive effects against gastric cancer cells. **a** In vitro wound-healing assay showed the suppressive effect of miR-612 on cell migration. **b** miR-612 overexpression significantly inhibited the invasion of both AGS and SGC-7901 cells. **c** Conditioned media from miR-612-overexpressing AGS and SGC-7901 cells showed a reduced angiogenic activity. **d** Western blot analysis of E-cadherin (E-cad) and vimentin (Vim) protein levels. ^*^P < 0.05 vs. control miR-transfected cells

Next, we checked the biological role of miR-612 in gastric cancer. In vitro wound-healing assay revealed that cell migration was restrained after miR-612 overexpression in both AGS and SGC-7901 cells (Fig. 5a). Consistently, the invasive ability of gastric cancer cells was reduced by miR-612 overexpression (Fig. 5b). Ectopic expression of miR-612 impaired the angiogenic activity of AGS and SGC-7901 cells (Fig. 5c). In addition, the level of E-cadherin was increased and vimentin was decreased in miR-612-overexpressing gastric cancer cells (Fig. 5d). These observations indicate miR-612 as a tumor suppressor in gastric cancer.

**Fig. 6:**
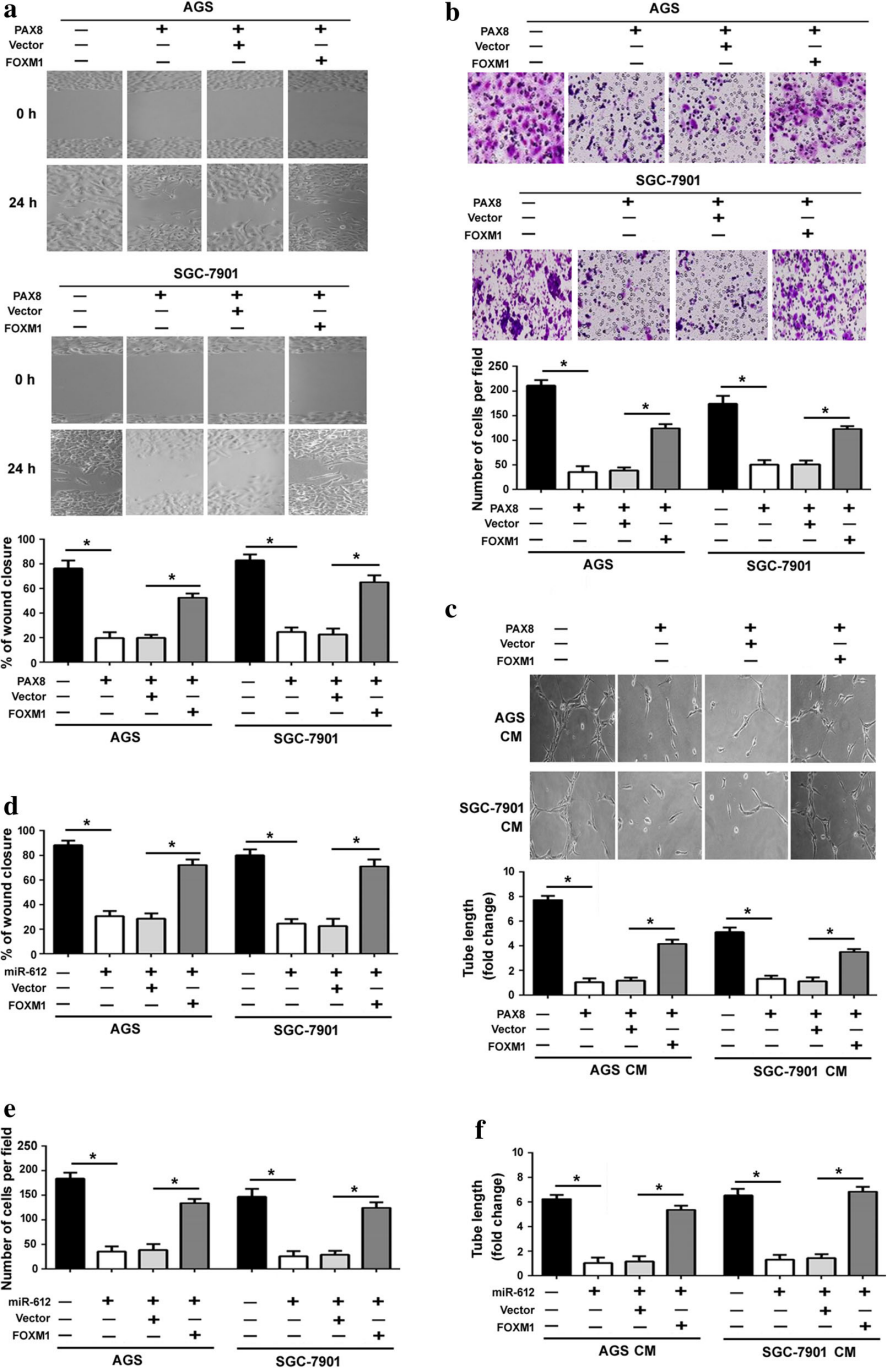
Overexpression of FOXM1 reverses the tumor-suppressive activity of PAX8 in gastric cancer cells. AGS and SGC-7901 cells were transfected with indicated constructs and tested for migration (**a** and **d**), invasion (**b** and **e**), and angiogenic activity (**c** and **f**). Bar graphs show the results from three independent experiments. ^*^P < 0.05

To validate the biological significance of miR-612/FOXM1 axis, we performed rescue experiments. The results showed that overexpression of FOXM1 significantly rescued gastric cancer cells from PAX8-mediated inhibition of cell migration (Fig. 6a) and invasion (Fig. 6b). Moreover, knockdown of miR-612 or overexpression of FOXM1 restored the angiogenic activity of gastric cancer cells with stable expression of PAX8 (Fig. 6c). Similarly, enforced expression of FOXM1 restored the aggressive phenotype of gastric cancer cells with miR-612 overexpression (Fig. 6d-f).

### PAX8 overexpression suppresses tumor angiogenesis and metastasis in nude mice

**Fig. 7:**
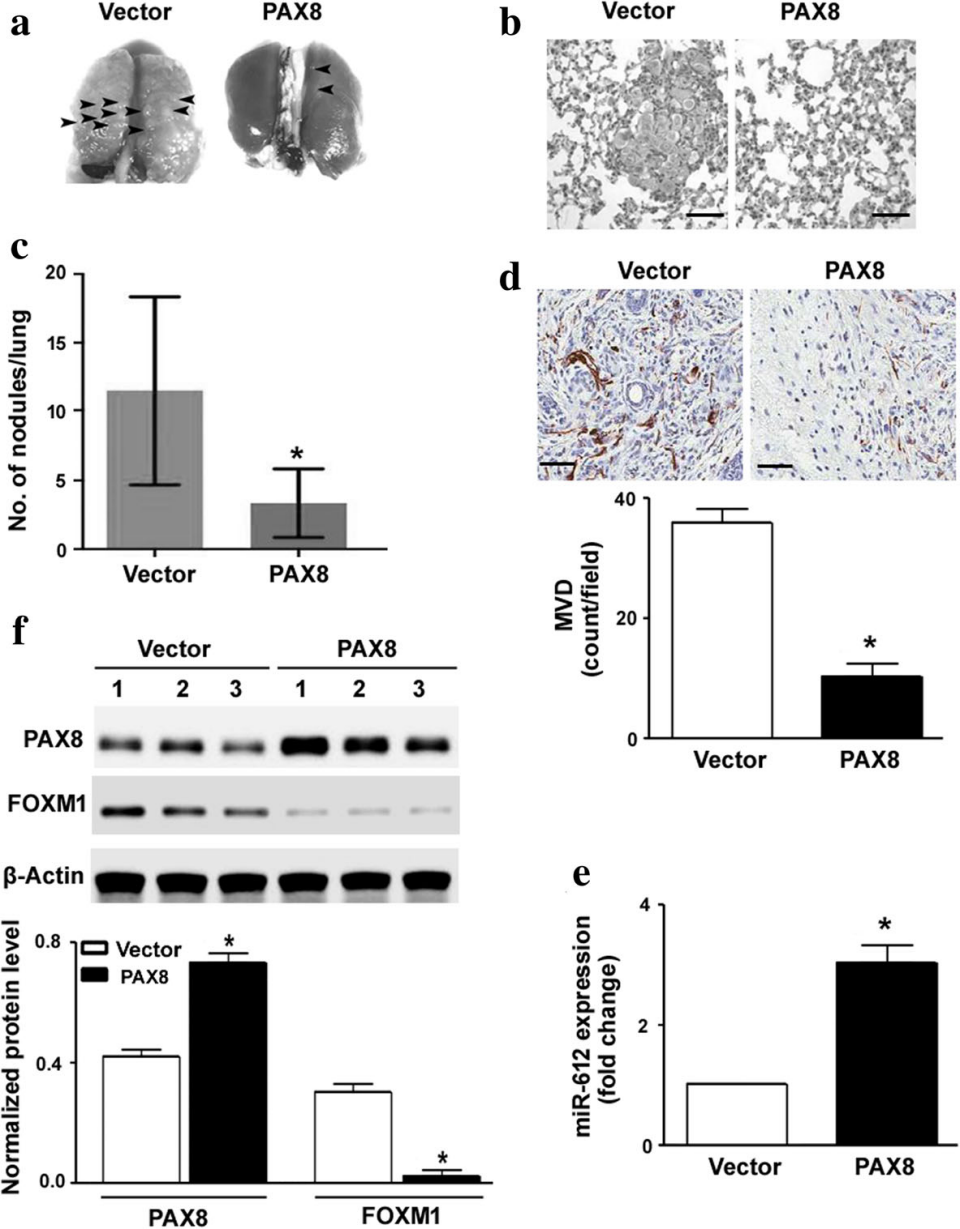
PAX8 overexpression suppressed tumor angiogenesis and metastasis in nude mice. SGC-7901 cells stably expressing PAX8 or empty vector were injected through the tail vein, and lung metastasis was assessed. **a** Representative photographs of the lung with metastatic lesions (arrowhead). **b** Representative H&E-stained lung tissue sections. Scale bar = 50 μm. **c** The number of metastatic lesions in the lung was determined (*n* = 6). **d** Analysis of tumor angiogenesis by CD31 immunostaining. PAX8 overexpression caused a reduction in CD31-positive MVD (*n* = 6). Scale bar = 80 μm. **e** Real-time PCR analysis of miR-612 levels in the lung tissue (*n* = 6). **f** Western blot analysis of FOXM1 and PAX8 protein levels in the lung tissue (*n* = 6). *Top*, representative blots from 1 to 3 mice are shown

Finally, we determined the effect of PAX8 overexpression on tumor metastasis in vivo. To this end, SGC-7901 cells stably expressing PAX8 or empty vector were injected through the tail vein. The number of metastatic lesions in the lung was significantly lower in the PAX8 overexpression group than in the control group (*P* < 0.05; Fig. 7a-c). Analysis of tumor angiogenesis by CD31 immunostaining revealed that PAX8 overexpression led to a significant decline in the CD31-positive MVD (Fig. 7d). In addition, the expression of miR-612 (Fig. 7e) was increased and FOXM1 (Fig. 7f) was decreased in the lung from the PAX8 overexpression group.

## Discussion

Our findings reveal the downregulation of PAX8 in gastric cancer cell lines relative to normal gastric epithelial cells, which is in line with a previous study showing decreased expression of PAX8 in gastric adenocarcinomas [12]. The mechanism for PAX8 downregulation in gastric cancer is currently unknown. Since the mRNA and protein levels of PAX8 consistently decreased in gastric cancer cells, this downregulation may occur at the transcriptional level.

Overexpression of PAX8 caused a significant suppression of gastric cancer cell migration and invasion, but had no significant impact on cell proliferation. Depletion of PAX8 facilitated the migration and invasion of gastric cancer cells. In vivo studies revealed that PAX8 overexpression restrained the metastatic activity of gastric cancer cells in nude mice, which was accompanied by compromised angiogenesis. These observations indicate an anti-metastatic activity for PAX8 in gastric cancer. However, in ovarian cancer, knockdown of PAX8 was found to reduce cancer cell proliferation, migration, and invasion [11]. Similarly, PAX8 overexpression enhances the migration and tumorigenicity of thyroid carcinoma cells [23]. Therefore, we speculate that PAX8-mediated signaling pathways are unique to specific cancer cell types.

Our findings furthermore demonstrate the suppression of EMT by PAX8. We found that PAX8-overexpressing gastric cancer cells displayed an upregulation of E-cadherin and downregulation of vimentin. The importance of EMT in gastric cancer development and progression has been well documented [24]. Inhibition of EMT is accompanied by reduced invasive ability in gastric cancer cells with overexpression of ERp29 [24]. Therefore, PAX8-mediated anti-invasive activity may be ascribed to prevention of EMT. In addition, ectopic expression of PAX8 interfered with the pro-angiogenic activity of gastric cancer cells, as determined by in vitro endothelial cell tube formation assay. VEGF expression and secretion was suppressed by PAX8, which provides an explanation for the reduced angiogenic activity in gastric cancer cells. Collectively, these results support the conclusion that PAX8 acts as a tumor suppressor in gastric cancer.

FOX proteins play a pivotal role in tumor growth and metastasis through transcriptional regulation of a number of cancer genes [25]. Our data showed that the expression of FOXM1 was significantly reduced in PAX8-overexpressing gastric cancer cells, but FOXC2, FOXF1, and FOXL1 remained unchanged. Overexpression of FOXM1 has been reported to promote gastric cancer cell invasion [15]. Knockdown of FOXM1 suppresses the EMT and expression of VEGF in gastric cancer cells [26], which resembles the phenotype observed in PAX8-overexpressing gastric cancer cells. Furthermore, enforced expression of FOXM1 reversed the anti-invasive activity of PAX8 in gastric cancer cells. These results collectively point toward that FOXM1 is a direct mediator of PAX8-induced tumor suppression. However, in terms of regulation of cell proliferation, PAX8 overexpression and FOXM1 depletion do not elicit the same response. In contrast to no effect with PAX8 overexpression, FOXM1 knockdown was reported to inhibit gastric cancer cell proliferation [26]. The discrepancy may be explained by the upregulation of growth-promoting genes, which compensates for the reduced proliferation caused by FOXM1 knockdown. This hypothesis needs to be validated in future work.

With regard to the molecular basis of PAX8-mediated downregulation of FOXM1, we focused on miRs that negatively regulate a large number of target genes. We predicted that several miRs including miR-612 targeted the 3’-UTR of FOXM1 mRNA. Most importantly, overexpression of miR-612 significantly suppressed the expression of FOXM1, suggesting that FOXM1 serves as a target of miR-612. This miR was selectively stimulated by PAX8 overexpression in gastric cancer cells. Depletion of miR-612 impaired the downregulation of FOXM1 by PAX8. Therefore, we provide evidence that PAX8-mediated reduction of FOXM1 in gastric cancer cells is ascribed to induction of miR-612. Functionally, knockdown of miR-612 reversed the tumor-suppressive activity of PAX8, which recapitulates the effect of FOXM1 overexpression on PAX8-overexpressing gastric cancer cells. In line with our findings, miR-612 was found to suppress the metastasis of hepatocellular carcinoma and colorectal cancer [27, 28]. These results indicate that miR-612 commonly functions as a tumor suppressor.

## Conclusion

Our work identifies PAX8 as a key negative component of the metastatic cascade in gastric cancer. By inducting the expression of miR-612, PAX8 is able to downregulate FOXM1 in gastric cancer cells, consequently leading to reduced cancer cell’s invasive, angiogenic, and metastatic potential. Our findings suggest that overexpression of PAX8 and/or miR-612 may represent a promising therapeutic strategy for gastric cancer.

## Abbreviations

3’-UTR: 3′-untranslated regions
ELISA: Enzyme-linked immunosorbent assay
EMT: Epithelial-mesenchymal transition
FOXM1: Forkhead box M1
HUVECs: Human umbilical vein endothelial cells
miR-612: MicroRNA (miR)-612
MTT: 3-(4,5-dimethylthiazol-2-yl)-2,5-diphenyltetrazolium bromide
MVD: Mean vessel density
PAX8: Paired box 8
siRNA: Small interfering RNA
VEGF: Vascular endothelial growth factor
